# Diagnosis of pulmonary adenoid cystic carcinoma using multimodal ultrasound imaging technology: A case report

**DOI:** 10.1097/MD.0000000000041212

**Published:** 2025-01-03

**Authors:** Hong Shi, Wei Qiu, Ping Yang, Yaping Zhang, Tingyu Huang, Jinyuan Mei, Xinrui Yin, Yanhui Yang

**Affiliations:** a Department of Ultrasound, The First People’s Hospital of Neijiang, Neijiang City, Sichuan Province, China; b Laboratory of Pathology, The First People’s Hospital of Neijiang, Neijiang City, Sichuan Province, China; c School of Clinical Medicine, Southwest Medical University, Luzhou City, Sichuan Province, China; d School of Clinical Medicine, Chengdu Medical College, Chengdu City, Sichuan Province, China; e Department of Thoracic Surgery, The First People’s Hospital of Neijiang, Neijiang City, Sichuan Province, China.

**Keywords:** contrast-enhanced ultrasound, interventional ultrasound, pulmonary adenoid cystic carcinoma, shear wave elastography

## Abstract

**Rationale:**

Pulmonary adenoid cystic carcinoma (PACC) is an exceedingly uncommon malignant tumor originating from salivary glands.

**Patient concerns:**

We present a case of primary PACC with multiple lung metastases in a 48-year-old male patient.

**Diagnosis:**

Diagnosis involved grayscale ultrasound, shear wave elastography, contrast-enhanced ultrasound, and ultrasound-guided percutaneous lung biopsy, all conducted in a one-stop manner, and confirmed by pathological examination.

**Interventions:**

Treatment was recommended after the diagnosis was confirmed, but was not accepted.

**Outcomes:**

With telephone follow-up, the patient survived.

**Lessons:**

PACC is an exceedingly uncommon malignant tumor originating from salivary glands. Compared to other cases, this case highlights the potential of multimodal ultrasound imaging for diagnosing lung tumors.

## 1. Introduction

Adenoid cystic carcinoma (ACC) originating from the lungs is rare, accounting for only 0.04% to 0.2% of lung cancer cases.^[1]^ ACC is a rare malignant tumor, belonging to the salivary gland type tumor, usually occurring in the salivary gland, can also be primary in the external auditory canal, esophagus, breast, lung, bronchial tract, and other organs.^[2,3]^ Previous studies have undertaken preliminary investigations into the adjunctive diagnosis of peripheral lung tumors using ultrasound elasticity technology.^[4]^ Furthermore, contrast-enhanced ultrasound (CEUS) enables real-time dynamic observation of tissue microvascular perfusion characteristics, furnishing crucial insights for the qualitative diagnosis of peripheral lung tumors.^[5]^ Ultrasound contrast-assisted guidance for percutaneous lung mass puncture facilitates improved differentiation between necrotic and active areas, thus enhancing puncture precision.^[6]^ This case report synthesizes the diagnostic efficacy of current ultrasound imaging modalities for this pathology.

## 2. Case presentation

**Figure 1.**
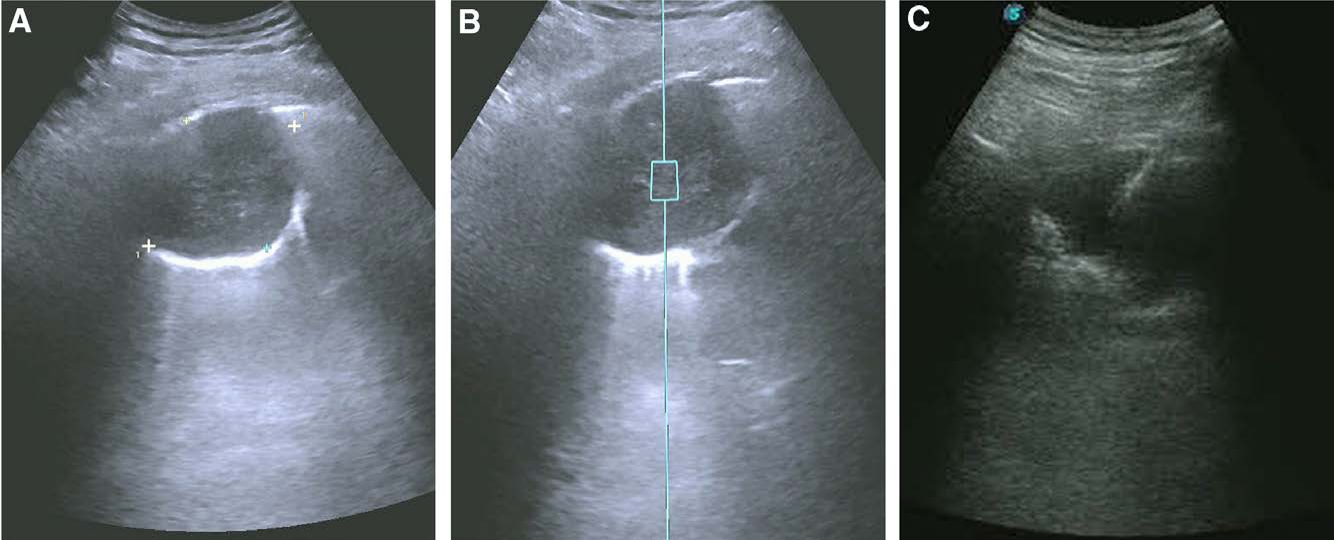
(A) Gray scale ultrasound indicates a hypoechoic mass under the right pleura, which is round in shape with unclear boundaries and uneven internal echoes. A “comet tail sign” can be seen behind the mass. (B) Shear wave elastography indicates an Emean of 7.9 kPa of the mass. (C) Ultrasound-guided mass puncture surgery.

**Figure 2.**
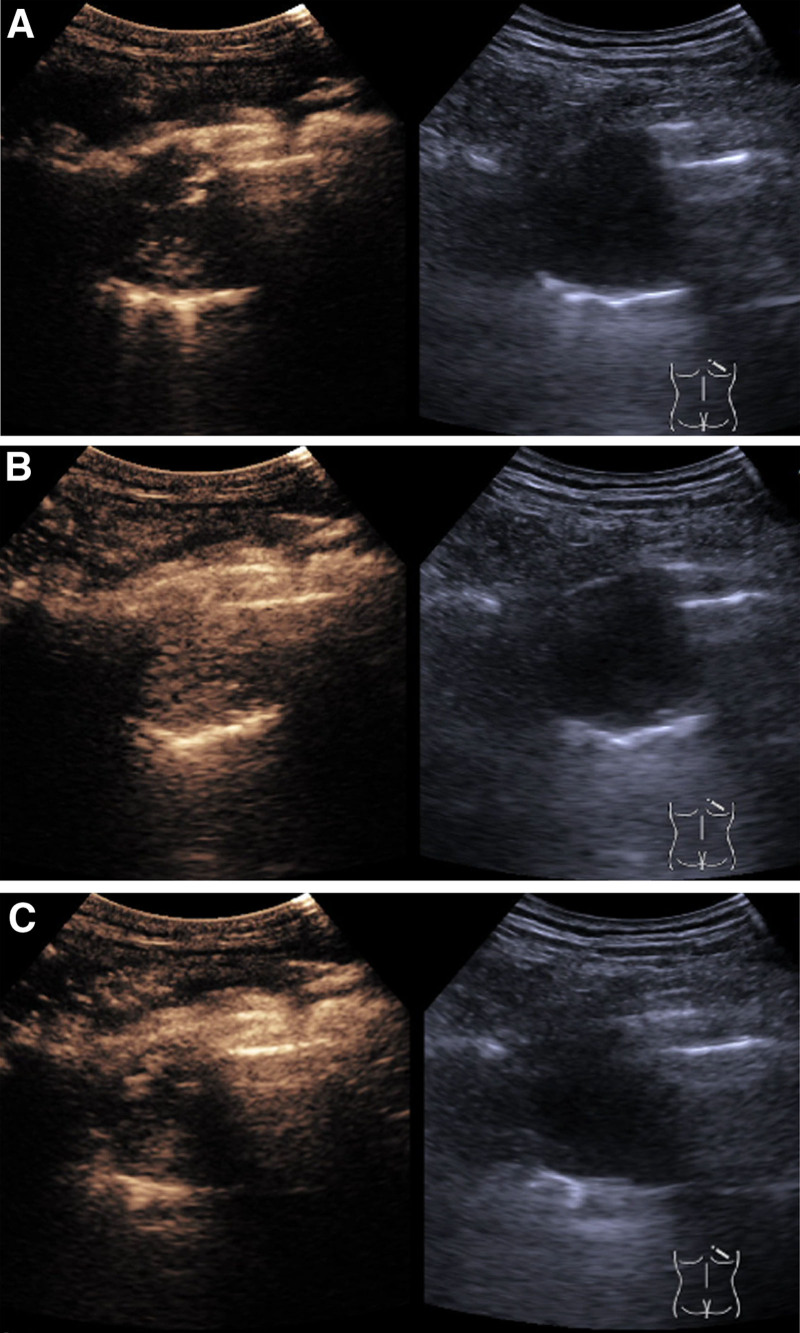
(A) Ultrasound contrast enhanced at 14 seconds, with thick and twisted vascular branches around the mass. (B) At 27 seconds, the enhancement reached its peak, with most of the masses showing high enhancement and some areas without enhancement visible inside. (C) At 1 minute and 12 seconds, the contrast agent gradually decreased and the mass showed uneven low enhancement.

We report the case of a 48-year-old male who was admitted to the hospital with a 4-month history of back and chest pain, as well as persistent cough and white, mucous-like sputum. The patient did not experience other symptoms such as shortness of breath, fever, chills, night sweats, or hoarseness. Physical examination and laboratory tests revealed no significant abnormalities. The patient has no history of hypertension or diabetes and no history of malignant tumors. No history of smoking or alcohol consumption. A Siemens Sequoia probe was utilized for color ultrasound examination. Gray scale ultrasound identified a hypoechoic mass beneath the right pleura (Fig. 1A), measuring approximately 6.3 × 4.7 cm. The mass appeared quasi-circular with indistinct boundaries and uneven internal echoes, exhibiting a comet tail sign posteriorly. Shear wave elastography (SWE) indicated a mean Young modulus value (Emean) of 7.9 kPa within the mass (Fig. 1B). CEUS demonstrated enhancement beginning at 14 seconds, with prominent, tortuous blood vessels penetrating the mass (Fig. 2A). Peak enhancement was observed at 27 seconds (Fig. 2B), followed by a gradual decrease starting at 1 minute and 12 seconds (Fig. 2C). Non-enhancing areas and irregular necrosis were evident within the mass. Ultrasound diagnosis indicated a right subpleural hypoechoic mass consistent with a lung tumor. Under ultrasound guidance, percutaneous puncture of the subpleural mass was performed (Fig. 1C), avoiding large blood vessel branches and necrotic regions. Three tissue samples were obtained for pathological examination.

**Figure 3.**
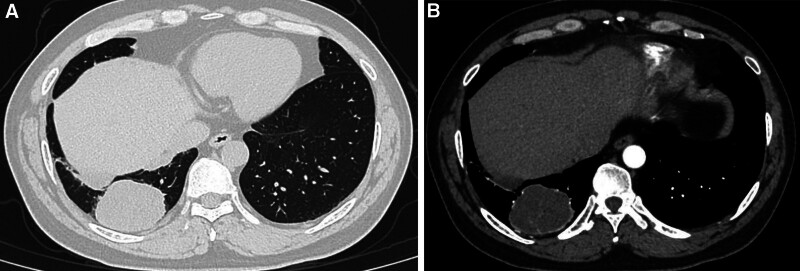
(A) CT plain scan reveals multiple clustered soft tissue density shadows in both lungs. (B) CT enhancement suggests mild to moderate continuous enhancement of the mass.

**Figure 4.**
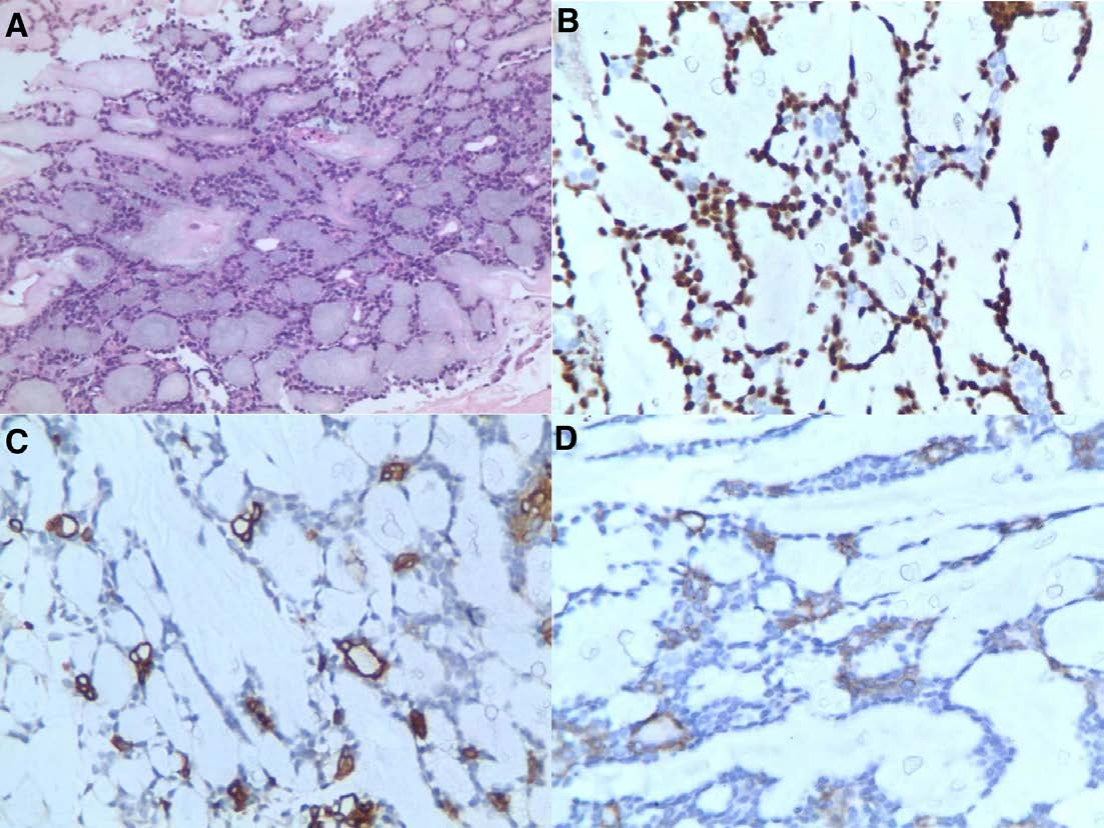
(A) Microscopy shows fibrous tissue proliferation and degeneration, with irregular cell nests visible in the stroma, presenting a sieve like and tubular structure (H&E staining, 100×). (B) P63 myoepithelium cells + (Immunohistochemistry, 400×). (C) CK7 luminal cells + (Immunohistochemistry, 400×). (D) CD117 inner layer cells + (Immunohistochemistry, 400×).

CT scans reveal multiple clustered soft tissue density shadows in both lungs (Fig. 3A), exhibiting mild to moderate continuous enhancement upon contrast-enhanced scans (Fig. 3B), indicative of lung tumors with widespread metastases. Pathological biopsy, alongside morphological and immunohistochemical findings (Fig. 4), supports the diagnosis of ACC of the lung.

## 3. Discussions

Pleomorphic adenoma carcinoma (PACC) arises from the submucosal glands of the trachea and bronchi, typically afflicting individuals aged 40 to 60 years. Clinical presentations of PACC are often nonspecific, with many patients initially presenting with an unexplained cough. Additional symptoms may include obstructive pneumonia, dyspnea, wheezing, and chest pain. The diagnosis of PACC necessitates a comprehensive evaluation encompassing clinical manifestations, imaging studies, immunohistochemistry, histopathology, and other pertinent factors.

Gray scale ultrasound reveals a “comet tail sign” trailing behind the lesion, a highly specific and sensitive indicator for detecting metastatic cancer. This sign arises from multiple reflections due to the substantial acoustic impedance disparity between the uniform metastatic lesion and the surrounding lung gas. In a study involving 95 patients with peripheral pulmonary lesions, researchers conducted elasticity tests and observed that lung cancer tissue exhibited significantly greater hardness (4.67 ± 0.49 kPa) compared to pneumonia tissue (2.35 ± 0.48 kPa).^[7]^ Ozgokce et al^[8]^ demonstrated the significance of SWE in discriminating between benign and malignant peripheral pulmonary lesions. An increase in mean Young modulus value (Emean), a quantitative indicator of SWE, suggests heightened tissue stiffness and reduced elasticity, indicative of malignant lesions.

Previous studies have demonstrated that benign lung lesions typically exhibit early and pronounced contrast enhancement on ultrasound imaging, suggesting a supply from the pulmonary artery. Conversely, malignant lesions often display delayed and weaker enhancement compared to benign lesions, indicating either a lack of pulmonary artery supply or blood provision from bronchial arteries.^[9]^ In 2017, the European Federation of Societies for Ultrasound in Medicine and Biology issued guidelines for CEUS, proposing that an enhancement onset time of <10 seconds suggests benign lesions, while an onset time exceeding 7.5 seconds indicates potential malignancy.^[6]^ In this instance, contrast agent infusion commenced relatively late, accompanied by the presence of thick and tortuous blood vessels supplying the mass and areas of non-enhanced necrosis: distinctive features of ultrasound contrast in pulmonary malignancies. Utilizing these imaging findings, ultrasound interventionists can select a more scientifically informed puncture trajectory to enhance puncture success rates. Ultrasound-guided percutaneous peripheral lung puncture offers advantages such as simplicity, minimal invasiveness, absence of radiation, and repeatability, with puncture success rates exceeding 80%, rendering it widely employed in clinical settings.^[10]^ As ultrasound technology advances, multimodal ultrasound diagnostic techniques are increasingly applied in the evaluation of peripheral lung masses to enhance disease diagnosis accuracy and supplement limitations of conventional radiation-based imaging modalities.

## Acknowledgments

Thank you to all team members for your dedication.

## Author contributions

**Conceptualization:** Jinyuan Mei, Yanhui Yang.

**Data curation:** Jinyuan Mei, Yanhui Yang.

**Formal analysis:** Jinyuan Mei, Yanhui Yang.

**Investigation:** Xinrui Yin, Yanhui Yang.

**Methodology:** Xinrui Yin, Yanhui Yang.

**Project administration:** Xinrui Yin, Yanhui Yang.

**Resources:** Tingyu Huang.

**Software:** Tingyu Huang.

**Supervision:** Tingyu Huang.

**Validation:** Tingyu Huang.

**Visualization:** Tingyu Huang.

**Writing – original draft:** Hong Shi, Wei Qiu, Ping Yang.

**Writing – review & editing:** Yaping Zhang.
